# A Spotlight on Viruses—Application of Click Chemistry to Visualize Virus-Cell Interactions

**DOI:** 10.3390/molecules24030481

**Published:** 2019-01-29

**Authors:** Thorsten G. Müller, Volkan Sakin, Barbara Müller

**Affiliations:** 1 Department of Infectious Diseases, Virology, University Hospital Heidelberg, 69120 Heidelberg, Germany; Thorsten.Mueller@med.uni-heidelberg.de; 2 Department of Infectious Diseases, Molecular Virology, University Hospital Heidelberg, 69120 Heidelberg, Germany; Volkan.Sakin@med.uni-heidelberg.de

**Keywords:** virus, fluorescence microscopy, bioorthogonal, click chemistry, amber suppression, single virus tracking, super-resolution microscopy, nucleoside analog, genetic code expansion, non-canonical amino acid, unnatural amino acid, metabolic labeling, de novo DNA labeling, de novo RNA labeling, DNA virus, RNA virus, retrovirus, replication compartments, viral factories, inclusion bodies, reverse transcription, uncoating, EdU, EU

## Abstract

The replication of a virus within its host cell involves numerous interactions between viral and cellular factors, which have to be tightly controlled in space and time. The intricate interplay between viral exploitation of cellular pathways and the intrinsic host defense mechanisms is difficult to unravel by traditional bulk approaches. In recent years, novel fluorescence microscopy techniques and single virus tracking have transformed the investigation of dynamic virus-host interactions. A prerequisite for the application of these imaging-based methods is the attachment of a fluorescent label to the structure of interest. However, their small size, limited coding capacity and multifunctional proteins render viruses particularly challenging targets for fluorescent labeling approaches. Click chemistry in conjunction with genetic code expansion provides virologists with a novel toolbox for site-specific, minimally invasive labeling of virion components, whose potential has just recently begun to be exploited. Here, we summarize recent achievements, current developments and future challenges for the labeling of viral nucleic acids, proteins, glycoproteins or lipids using click chemistry in order to study dynamic processes in virus-cell interactions.

## 1. Introduction

**Figure 1:**
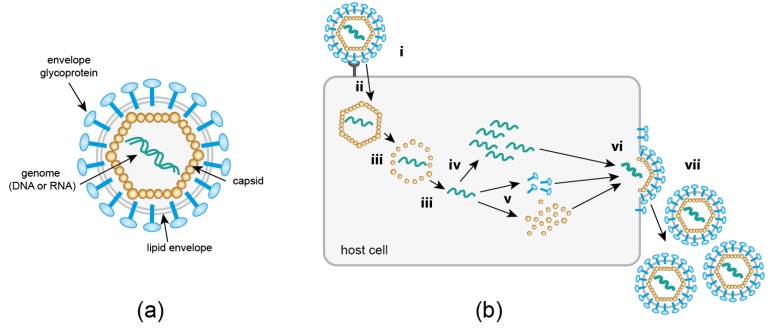
(**a**) Basic components of a virus particle. Non-enveloped viruses consist of nucleic acid encased in a proteinaceous capsid. Enveloped viruses are further surrounded by a lipid envelope derived from a host cell membrane, studded with viral glycoproteins required for cell attachment and entry. (**b**) Generic replication cycle of eukaryotic viruses. Details of individual steps vary greatly between different virus families. Receptor mediated attachment (i) is followed by cytosolic entry (ii), which may occur via an endocytotic process or—for enveloped viruses—membrane fusion. An uncoating step (iii) releases the viral genome, which is replicated (iv) either in the cytosol or in the nucleus of the host cell. Viral proteins are produced by the host cell translation machinery (v). Finally, newly synthesized virion components assemble into progeny particles (vi); this may occur either within the cell or directly underneath the plasma membrane. Release (vii) of progeny virus occurs via cell lysis, exocytosis or budding at the cell membrane.

Viruses occupy a unique position at the boundary of life. Outside of their host, they exist as (largely) inert macromolecular assemblies, which awaken to a “borrowed life” upon entry into a suitable host cell. A virus particle, or virion (Figure 1a), essentially consists of genomic information enclosed in a protective protein shell (capsid). In some cases (“enveloped viruses”), the capsid is further surrounded by a lipid membrane derived from the host cell. Although viruses may additionally encode enzymes or accessory proteins to promote their replication and spread, they lack the autonomous machineries required for protein synthesis and metabolism. Therefore, viruses represent obligatory pathogens which can only replicate by usurping metabolic resources, trafficking pathways and enzymatic machineries of their host. This exploitation of cellular functions, as well as cellular defense strategies directed against the invading pathogen, involves numerous dynamic, complex, and highly regulated interactions between viral and cellular components. Understanding the intricate interactions between a virus and its host cell is not only highly relevant for identifying weaknesses of pathogenic viruses that may serve as targets for antiviral strategies, but can also provide unique insights into the cellular machinery (ab)used by the intruder. Since a number of virus particles can enter a single host cell—potentially following different productive pathways—and a large number of progeny particles are produced in an asynchronous manner, single virus imaging and real-time single virus tracking (SVT) approaches have proven to be crucial for the investigation of virus-host cell relationships.

The past twenty years have brought a dramatic shift in the field of virus imaging. For decades, researchers had to rely on negative staining or thin section electron microscopy (EM), or x-ray crystallography to visualize the minute pathogens. Since these approaches depend on the detection of morphologically recognizable structures, or on crystals of purified material, respectively, they are mainly restricted to the analysis of free extracellular virions, or of particles attached to or being released from a host cell. A substantial part of the viral life cycle (Figure 1b) escapes this type of observation. A feature that distinguishes viruses from all organisms is that their replication comprises a so-called ‘eclipse’ phase. The entering virion undergoes a disassembly process during which particle integrity is lost; only the genomic information of the virus is retained in the infected cell. The genome serves as the basis for the synthesis of new virion components, which are subsequently assembled into progeny particles. As a consequence, the composition and shape of “the virus” undergoes continuous transformation throughout the replication cycle, making it difficult or impossible to identify subviral structures within the crowded host cell environment by EM. This problem is being addressed by currently emerging correlative light and electron microscopy (CLEM) approaches, which aim at resolving structures of intracellular subviral complexes by electron tomography or focused ion beam milling–scanning EM, guided by initial fluorescence-based identification of objects of interest. Like all EM techniques, however, CLEM yields snapshots rather than capturing the dynamic information essential for a full understanding of the infection process.

A key technology for the observation of dynamic processes within living cells is fluorescence microscopy. Although the size of most virus particles, with diameters in the range of 20 to 300 nm, is below the resolution limit of light microscopy, the attachment of a fluorescent label makes it possible to identify and track single virus particles or subviral complexes within the infected cell. Laser scanning confocal microscopy and spinning disc confocal microscopy enable the 3D tracking of objects over time with high temporal resolution; total internal reflection microscopy focuses on events occurring at the plasma membrane; and light sheet microscopy makes it possible to image viral spread in tissue samples or organoids [1,2]. In recent years, super-resolution microscopy like structured illumination microscopy (SIM), single molecule localization microscopy (SMLM) techniques such as PALM/(d)STORM and stimulated emission depletion (STED) nanoscopy have lowered the effective resolution of fluorescence microscopy to the level of subviral structures [2,3], and thereby, greatly expanded the possibilities for detailed investigation of virus-cell interactions (for a recent in-depth review on these methods see [4]). Initially, nanoscopic techniques were mainly restricted to 2D analysis of fixed samples in one or a few color channels, but rapid technological development in this field has already begun to overcome these limitations, and substantial progress can be expected in the coming years. In addition, increasing the throughput of advanced imaging data acquisition and growing information density requires sophisticated analysis pipelines for objective and quantitative analyses of imaging data, computational modeling of structures derived from SRFM (e.g., [5]), and 4D tracking of the complex mobility of individual virus particles in noisy live-cell imaging sequences (reviewed in [1,6]). 

In order to make use of advanced microscopy methods, a fluorescent label needs to be attached to the molecule of interest. For the labeling of proteins, the genetically encoded marker green fluorescent protein (GFP) and the constantly expanding panel of engineered fluorescent proteins (FPs) [7] have been, and still are, invaluable tools for the investigation of dynamic events in cell biology. However, besides their relatively high molecular mass of ~ 27 kDa, FPs have clear limitations regarding the use of advanced light microscopic methods and the study of viruses in general [8]. Advanced fluorescence microscopy methods have specific requirements regarding the photophysical properties of the fluorophore, which are often not met by FPs. Single virus tracking within an infected cell requires high fluorescence intensity of the label for sensitive and quantitative detection of individual particles, and high photostability of the dye to allow for the acquisition of image sequences with high temporal resolution. Furthermore, the relatively slow maturation of the fluorophore characteristic for most fluorescent proteins prevents the observation of tagged proteins shortly after translation, and pH-dependent quenching may obscure signals in the endosomal compartment. STED and PALM/STORM rely on fluorophores that can switch between an ‘on’ and ‘off’ state, and the high laser intensities required for emission depletion in standard STED microscopy again requires highly photostable labels. All these requirements are fulfilled by synthetic dyes, which also provide a large variation of excitation/emission maxima for multi-color imaging, including dyes in the far-red range.

The advancement of micro- and nanoscopic methods is therefore paralleled by continuous development of novel fluorescent dyes with properties tailored to the requirements of specific applications. Stainable genetically encoded tags, e.g., SNAP-, Halo-, TMP- or FAST-tag [9] allow the attachment of synthetic fluorophores to a protein of interest (POI). However, the molecular dimensions of these tags are not negligible compared to the size of the POI. While this is a general concern for any POI to be tagged, viral proteins are particularly challenging targets for genetic modification. Many viruses are characterized by small genomes with overlapping open reading frames and increasing the genome size may affect its packaging into the viral capsid. Viral structural proteins are typically small and multifunctional, and they need to assemble into regular and stable multimeric structures. Both properties result in low tolerance towards structural alterations. Therefore, minimally invasive labeling strategies are of particular importance for virus imaging.

**Figure 2:**
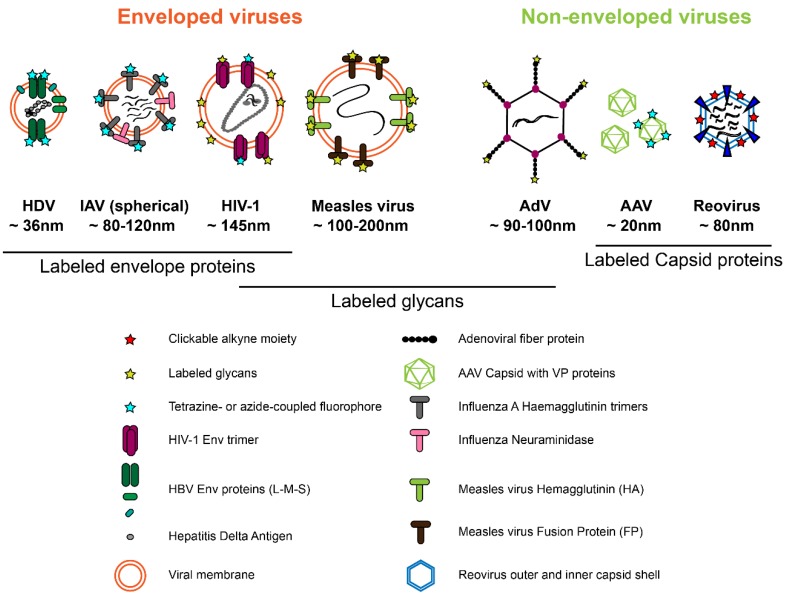
Viral proteins that have been successfully modified by click labeling strategies. See Section 2 for details.

Traditionally, labeling of (mainly non-enveloped) viruses has been performed by covalent attachment of chemically reactive fluorophores to the primary amine group of lysines or free sulfhydryl groups of cysteine residues. However, in this case labeling is not restricted to a specific site, and its degree is difficult to control. The incorporation of a functionalized non-canonical amino acid (ncAA) into the POI via genetic code expansion (GCE), followed by the attachment of a synthetic fluorophore via a fast and specific bioorthogonal click reaction represents the most minimally invasive strategy for site-specific protein labeling [10,11,12]. The methodology had been originally established in bacteria. Its more recent adaptation to eukaryotic cells provides virologists with novel opportunities for site-specific introduction of a fluorophore of choice into their viral POI (Figure 2). While conceptually ideal for virus labeling, the approach still remains technically challenging, and its full potential for virus imaging has not yet been realized. Very recently, however, its application has begun to transcend from proof of principle studies to analyses yielding exciting new insights into virus biology. The modification of viral proteins with clickable groups and GCE also provides new options to target viral vectors to specific cell types, to engineer attenuated antiviral vaccines, to photoactivate virus functions, to immobilize viruses for physical measurements, or to mimic post-translational modifications. While these options are also extensively explored (e.g., [13,14,15,16]; see also [17,18]) we will focus here on the use of click chemistry for virus imaging.

Beyond site-specific protein modification, click chemistry has also provided virologists with tools for labeling other key viral components, most importantly nucleic acids. While the incorporation of clickable bioorthogonal nucleotide analogs is inherently nonspecific, the biology of viruses can be exploited to render nucleic acid labeling virus specific. Unlike the host genome, which is dsDNA by default, viral genomes come in different forms [19]. They may consist of DNA or RNA, single- or double-stranded, segmented or a single molecule. Furthermore, viruses employ replication strategies and/or sites not used by the host cell. For example, the replication cycle of retroviruses involves synthesis of dsDNA outside of the nucleus, and many RNA viruses replicate their genome in distinct virus-induced factories in the cytosolic region. Clickable nucleotide analogs could also be designed as substrates preferentially used by a virus encoded DNA or RNA polymerase. Finally, viruses carrying labeled genomes may be purified after release from a producer cell and then used to infect unlabeled host cells. Fluorescent labeling of lipids at virus assembly sites or in the virus lipid envelope, or of sugar moieties of viral glycoproteins also greatly benefits from click chemistry. In this review, we summarize concepts, current achievements and future challenges in the use of click chemistry for investigating virus-host cell interactions by advanced imaging approaches.

## 2. Minimally Invasive Labeling of Viral Proteins Using Click Chemistry

### 2.1. Metabolic Labeling of Viral Components

A straightforward approach for introducing bioorthogonal functional groups into viruses is the metabolic incorporation of functionalized building blocks for proteins, glycans or lipids that are components of the viral particle. While this incorporation will also affect components of the virus-producing cell, engineered particles may be purified from the tissue culture supernatant before fluorescent labeling. 

#### 2.1.1. Glycans

Like cellular proteins, viral proteins may be modified by glycosylation; in particular envelope proteins are often heavily glycosylated to avoid recognition by the immune system. The first example of labeling of the non-enveloped capsid of adenovirus (AdV) via click chemistry was accomplished for human adenovirus 5, targeting the sugar moiety of the fiber protein. The fibers form spikes associated with each penton base of the icosahedral capsid shell and carry a single *O*-linked *N*-acetylglucosamine (*O*-GlcNAc) on a serine residue. Banerjee et al. [13] replaced this natural sugar with azido derivatives such as tetraacetylated *N*-azidoacetyl-glucosamine or -galactosamine (Ac4GlcNAz, Ac4GalNAz) through metabolic incorporation. Subsequent copper-catalyzed azide-alkyne cycloaddition (CuAAC) mediated ligation to alkyne-coupled fluorophores or peptides demonstrated that AdV particles can be decorated with different functional groups through this sugar moiety without affecting virus production or infectivity. The resulting engineered virions were not used for imaging studies, but were decorated with molecules that mediate retargeting of the virus to a desired host cell type [13]. 

#### 2.1.2. Lipids

The lipid envelope encasing the capsid is a key feature of enveloped viruses. The envelope is derived from a host cell membrane; however, its lipid composition can differ from the parental membrane, since viruses target or create specific membrane microenvironments to promote virus assembly and budding. Membrane composition may also determine the fusion properties of the particle in the viral entry process. Lipid-binding proteins tagged with FPs and fluorescently labeled lipid derivatives have been employed to analyze the distribution of lipids in membranes at the nanoscale, but both manipulations may significantly influence the properties of the lipid under investigation. Metabolic labeling using clickable mimetics of lipids or lipid precursors [20] provides an elegant solution increasingly used in cell biology, but not yet really exploited in virology. Proof of concept was provided by introducing azide-choline into the membrane of vaccinia and influenza virus and demonstrating that the viral particles could be click labeled via strain-promoted azide-alkyne cycloaddition (SPAAC) [21].

#### 2.1.3. Proteins

Residue-specific modification of proteomes can be achieved by incorporation of the ncAA azidohomoalanine, an analog of methionine. This strategy has been explored for bacteriophages, and has also been used for the modification of AdV-derived gene therapy vectors (again with the aim of vector retargeting) without impairing vector production or infectivity [22]. However, this method is neither protein- nor site-specific, defying the purpose of minimally invasive, virus specific labeling. It also does not allow precise positioning of labels for single-molecule approaches. Recent studies have therefore explored the technically more challenging approach of GCE for incorporation of single modified amino acid residues. 

### 2.2. Labeling of Viral Proteins Using Genetic Code Expansion and Click Chemistry

**Figure 3:**
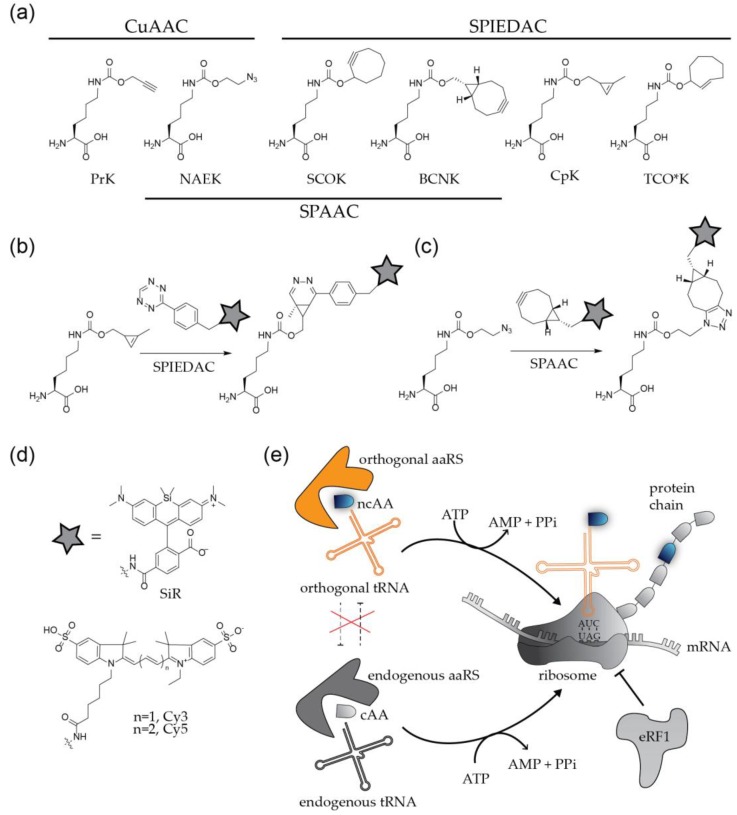
Labeling of proteins by genetic code expansion and click chemistry. (**a**) Structures of non-canonical amino acids frequently used for protein modification. (**b**,**c**) Coupling of functionalized fluorophores to bioorthogonal amino acids via strain promoted cycloadditions. (**d**) Organic fluorophores frequently applied in virological studies. (**e**) Principle of site-specific incorporation of ncAAs by amber suppression.

The site-specific engineering of proteins via click chemistry is a two-step process (Figure 3). In the first step, a bio-orthogonal amino acid carrying a chemically reactive group (Figure 3a) is incorporated at the position of interest by GCE. The second step comprises the click labeling reaction, which results in the covalent ligation of proteins with synthetic fluorophores or other molecules of choice (Figure 3b–d).

To apply this method to the modification of a viral POI, the codon at the position of choice needs to be replaced by an alternative codon that specifies the ncAA. In addition, a bioorthogonal tRNA recognizing this codon and a cognate aminoacyl-tRNA synthetase (aaRS) to charge the tRNA with the ncAA have to be expressed in the virus producing cell. The most widely used GCE approaches employ a natural stop codon to represent the ncAA [10,11,12]. While all three nonsense codons were successfully used for the incorporation of ncAAs, the amber stop codon (UAG) is preferred over ochre (UAA) and opal (UGA), due to its lower abundance in both prokaryotic and eukaryotic mRNAs. The method is therefore referred to as ‘amber suppression’. Amber stop codons are recognized and decoded as the 22nd amino acid pyrrolysine (Pyl) [23] in certain methanogenic archaea, which have served as the basis for developing suitable orthogonal tRNAPyl/aaRSPyl pairs that mediate specific incorporation of the ncAA of choice. The most commonly used tRNAPyl/aaRSPyl pairs are adapted from the archaea *Methanosarcina mazei* and *Methanosarcina barkeri* [10,11,12]. In practice, the gene of interest is mutated in the viral context, or in the context of a separately expressed viral protein, introducing a TAG stop codon at a position of choice. The modified virus or viral protein is then produced in cells that express the components of the suppression system (usually accomplished via transient transfection with expression constructs encoding the orthogonal tRNA/aaRS pair) in the presence of a ncAA added to the growth medium at high concentration (up to 1 mM). Successful amber suppression results in production of the full-length POI with a click-reactive chemical handle at the desired position (Figure 3e).

In a subsequent step, the engineered protein is conjugated via click reaction to a molecule of interest. Depending on the biological question to be addressed, this reaction will be performed either directly in the virus (or viral protein) producing cell, or upon purification of the engineered virions from the tissue culture supernatant. The chemical nature of both, the ncAA and the conjugated molecule has to be tailored to the planned application. More than 100 ncAAs with different sizes and different reactive groups have been described (examples in Figure 3a) [10]. Derivatives carrying the small linear alkyne group react efficiently with molecules functionalized with an azide group via CuAAC. This well established and efficient reaction has the disadvantage that the Cu(I) required for catalysis is highly toxic for bacterial and eukaryotic cells, limiting the technique to fixed samples. Copper-independent alternatives employ ncAAs carrying strained alkynes or alkenes such as cyclooctyne or trans-cyclooctene, which are highly reactive under live-cell conditions. Strained alkynes can react both with azide- or tetrazine-coupled organic fluorophores in a strain-promoted Huisgen-type 1,3 dipolar [3 + 2]-cycloaddition (SPAAC) reaction or strain-promoted inverse electron demand Diels-Alder reaction (SPIEDAC), respectively, whereas strained alkenes react only with tetrazines via SPIEDAC reaction (Figure 3b). Cyclooctyne and trans-cyclooctene carrying ncAAs can thus be orthogonal to each other. The SPAAC reaction is slow, with rate constants in the range of ~ 0.1–1 M^−1^s^−1^ whereas SPIEDAC reactions occur with rates of 10^2^–10^4^ M^−1^s^−1^, promoting rapid labeling reactions.

A wide selection of clickable molecules functionalized with azides or tetrazines is available. A frequently used molecule is functionalized biotin, preparing the POI for pull-down experiments with streptavidin-coated beads. For imaging purposes, the molecule of choice is an organic fluorophore (Figure 3d). The sensitive detection of viruses requires bright and photostable dyes, and special applications may introduce further constraints. Depending on the viral POI, SVT approaches may require cell or membrane permeable dyes; it may also be necessary to use a fluorogenic probe to reduce cellular background. Advanced super-resolution microscopy approaches as PALM/STORM or STED require blinking, photoswitchable or extremely photostable fluorophores [2,3]. A key advantage of GCE/click labeling over other protein labeling techniques is versatility: any suitable fluorophore can be rapidly integrated into the experimental approach. 

### 2.3. Application of Genetic Code Expansion and Click Labeling in Virology

#### 2.3.1. Non-Enveloped Viruses

Non-enveloped viruses consist of a genome enclosed in a proteinaceous capsid built from multiple copies of one or more capsid proteins. By default, capsid proteins need to assemble into regular and stable multimeric structures. Furthermore, they undergo crucial molecular interactions with the viral genome and possibly other virion components in the assembly process, as well as with host cell dependency and restriction factors in the steps of virion uptake and uncoating [24]. These multiple requirements strictly limit the possibilities for genetic modification of capsid proteins without interfering with virus particle formation or infectivity. Chemical modification by click chemistry provides an elegant solution for targeting difficult POI. For many non-enveloped viruses, the availability of high resolution structures of the virion, as well as often extensive data from site-directed mutagenesis experiments, can guide the selection of suitable sites for targeted modification. On the other hand, non-enveloped viruses have frequently been found to tolerate a limited degree of non-specific chemical modification of free amide- or sulfhydryl groups on the viral surface; thus, examples for the use of GCE/click chemistry for SVT purposes are still limited for this virus group.

The most frequently targeted representatives of this group are Adeno-associated viruses (AAV), small (~ 25 nm) icosahedral ssDNA viruses which infect humans and other primate species. Their apparent lack of pathogenicity and their ability to infect dividing and non-dividing cells, resulting in persistent gene expression, render AAV-derived viral delivery vectors particularly attractive candidate vehicles for gene therapy. A detailed characterization of AAV-host cell interactions in the early phases of the replication cycle is important to understand the process of gene transduction by AAVs and for development of improved AAV-based vectors. The labeling of AAV for imaging analyses of viral entry has been approached by fusion of eGFP to VP2, one of the three capsid proteins of AAV [25], or by carbodiimide coupling of quantum dots to reactive cysteines and lysines on the viral surface [26]. However, these strategies can affect virus infectivity, and the addition of quantum dots nearly doubles the hydrodynamic radius of the virion [26]. Proof of principle for minimally-invasive labeling of AAV was provided by Kelemen et al. [27], who showed that a region present in all three capsid proteins (VP1-3) of AAV2 can be engineered to incorporate an azido-lysine (AzK) residue without affecting virus infectivity. The resulting virus particles carried 60 reactive ncAA, which could be ligated via SPAAC to a strained alkyne-fluorophore conjugate; the virus was not used for imaging purposes in this study, however. Recently, Zhang et al. [28] identified positions in the VP1 of AAV2 that tolerate the incorporation of *N*ε-2-azideoethyloxycarbonyl-l-lysine (NAEK) (Figure 3a) and click labeling, and used the resulting viral particles in imaging experiments. Entering virions could be tracked through the endosomal pathway, and impaired transport of particles to the nucleus upon disruption of the actin cytoskeleton by cytochalasin B was visualized [28]. 

#### 2.3.2. Enveloped Viruses

The protein capsids or nucleoprotein complexes of enveloped viruses are surrounded by a phospholipid bilayer, which carries viral envelope glycoproteins required for virus attachment and entry as well as cellular transmembrane proteins. In principle, envelope proteins and lipids, as well as inner capsid proteins, of these viruses are interesting targets for fluorescent labeling. Studies published to date, however, focus on the envelope proteins which are (i) surface exposed, (ii) often not arranged in regular lattices and (iii) often highly glycosylated, opening the possibility for metabolic labeling. 

##### Metabolic Labeling of Glycosylated Viral Proteins

**Figure 4:**
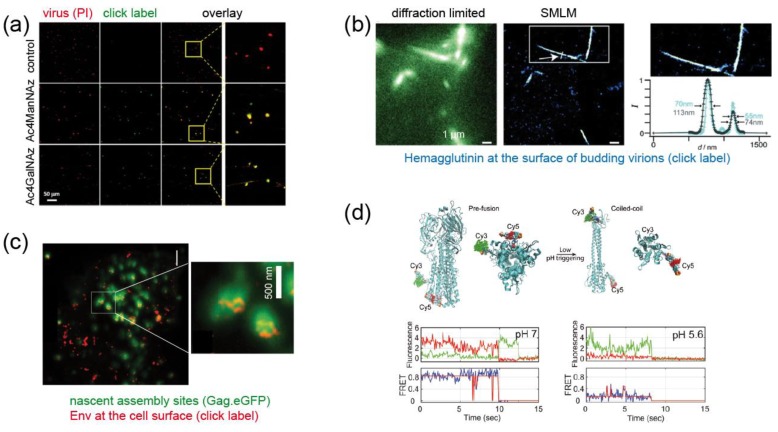
Examples for fluorescence labeling of viral proteins using click chemistry. (**a**) Metabolically labeled MV particles. The indicated sugars were added to the growth medium, virions were prepared and subjected to click labeling (green); propidium iodine staining (red) was performed to visualize all virions [29]. (**b**) Pulse-labeling of IAV HA exposed at the surface of budding virions using Cy3 and Cy5. The figure shows images of filamentous virus-like particles budding from the plasma membrane of a producer cell. Co-localization of both dyes at the nanoscale indicates incorporation of HA molecules synthesized at different times into the same particle [31]. (**c**) STED nanoscopy of click labeled HIV-1 Env in virus expressing cells shows clusters of Env molecules (red) recruited to viral assembly sites (visualized in diffraction limited mode via the structural protein Gag.eGFP; green) at the plasma membrane [32]. (**d**) Dual labeling of IAV HA at two different sites and smFRET reveal distinct conformational states of the molecule. Top, model of the modified protein in pre- and post-fusion conformation; bottom, smFRET traces recorded at pH conditions favoring the pre- (pH 7) and post-fusion (pH 5.6) state, respectively [33]. Images were modified from the indicated references, with permission.

A straightforward and efficient way of labeling the envelope of virions utilizes the fact that clickable azido sugar derivatives can be used by cellular glycosylation machineries instead of natural sugars without overt cytotoxic effects. Azido sugars added to the growth medium of cells are incorporated into glycoproteins, including viral envelope glycoproteins, and are subsequently labeled with alkyne-coupled fluorophores or other molecules of choice. The first example of envelope glycoprotein labeling via metabolic incorporation of clickable sugars was presented for the measles virus (MV), a (−)strand RNA virus belonging to the family of *Paramyxoviridae* [29]. The surface of the virion is decorated with hemagglutinin (HA), which mediates cell attachment, and with the viral fusion protein FP. Zhao et al. showed that *N*-azidoacetylmannosamine (Ac4ManNAz) and Ac4GalNAz can be metabolically incorporated into HA and FP and subsequently be click-labeled with alkyne-coupled fluorophores via SPAAC (Figure 4a) [29]. Although the labeled particles were not used to investigate the virus entry process, this study set the stage for the metabolic labeling of enveloped viruses. Oum et al. [30] adapted the strategy to the highly-glycosylated envelope (Env) protein of the retrovirus human immunodeficiency virus type 1 (HIV-1). Double-labeled particles were prepared by combining mCherry as a viral content marker with click labeling of Ac4ManNAz incorporated into the Env glycoprotein in order to differentiate intact virions from subviral particles that had undergone membrane fusion. This approach made possible the visualization of cytosolic entry events by SVT based on the separation of the envelope signal from the viral content [30]. 

##### Labeling of Viral Proteins by Amber Suppression

The metabolic incorporation of clickable sugars is straightforward, robust and relatively easy to perform, since it only requires addition of the compound to the growth medium. However, it is neither protein- nor site-specific, and can only be applied to glycoproteins. Therefore, the site-specific introduction of ncAA into envelope proteins by GCE has gained increasing interest in recent years. The first proof of concept came for the hepatitis delta virus (HDV), a satellite virus of human hepatitis B virus (HBV) and a major human pathogen. HDV is a small (~ 35 nm) spherical enveloped virusoid. Its circular ssRNA genome is the smallest mammalian virus genome (~ 1.7 kb). For replication, HDV depends on co-infection of the cell with HBV, whose large envelope protein (L-protein) is usurped by HDV to package its genome. Lin et al. [34] were able to incorporate several ncAAs with differing functionalities site-specifically into the HBV L-protein to produce engineered HDV particles. When the ncAA was introduced at a suitable position, particle yield and infectivity was comparable to wild-type HDV. Fluorophores or biotin molecules were clicked to the surface of engineered HDV by CuAAC [34], but the particles were not used for further biological studies. 

The influenza A virus (IAV), another important human pathogen, is a segmented (–)strand RNA virus of the genus *Orthomyxovirus*. The lipid envelope of influenza viruses is decorated with the viral fusion protein hemagglutinin (HA) and with neuraminidase (N) required for virus release. Nikic et al. [31] engineered influenza HA protein and established a pulse-chase strategy based on introducing TCOK* and SCOK as ncAA, followed by orthogonal labeling of these residues with Me-Tet-Cy5 and H-Tet-Atto532, respectively. Dual-labeled filamentous protrusions were visualized emerging from the plasma membrane of virus-producing cells by SMLM, demonstrating that HA-molecules synthesized at different times were incorporated into the same virus particle (Figure 4b) [31]. 

The application of GCE was further expanded to the Env subunit gp120 of HIV-1. Sakin et al. [32] showed for the first time that gp120 of HIV-1 accepts introduction of a ncAA residue at several positions within a variable surface loop of the protein without impairing its fusogenic activity; the modification was also compatible with virus infectivity. Click labeling of the engineered protein allowed the application of fluorescence recovery after photobleaching to analyze Env mobility at the plasma membrane of producing cells, which is relevant for the incorporation of the glycoprotein into nascent virus particles. The labeled protein was also shown to be suitable for STED nanoscopy, which permits the analysis of the nanoscale clustering of Env around viral assembly sites at the plasma membrane (Figure 4c) [32]. 

While these examples provide unique and important tools that hold promise for the future, a recently published, very elegant study raised the application of GCE/click labeling in virology to the next level. Das et al. [33] were interested in the conformational dynamics of the IAV HA protein, which is mechanistically relevant for promoting virus-cell fusion. In order to monitor the conformational state of the molecule in real time, they developed a single-molecule Förster resonance energy transfer (smFRET)-based approach to distinguish between the pre- and post-fusion conformation of the HA molecule. Pushing the current limits of the GCE technology, the authors simultaneously incorporated two TCOK* residues at strategic positions within the HA protein of IAV. SPIEDAC mediated click labeling with a FRET pair of different dyes (Cy3- and Cy5-tetrazine) yielded a high FRET signal for HA in the pre-fusion conformation, which was lost when the protein assumed its coiled-coil post fusion conformation (Figure 4d) [33]. Probing HA conformation under different conditions, the authors could show that the molecules are highly dynamic, reversibly interconverting between three distinct conformational states. Acidification (as it occurs during endosomal entry of the virus) and receptor binding shifted the conformational equilibrium, and additional membrane contact triggered irreversible conversion to the post-fusion state [33]. In this case, the exploitation of GCE/click labeling, combined with single-molecule imaging, yielded biological information that could not have been obtained by any other approach.

### 2.4. Virus-Specific Challenges and Limitations of GCE/Click Labeling and Possible Solutions

#### 2.4.1. Choice of Position

While it is clear that context-specific differences in suppression efficiency exist, the underlying mechanisms are still poorly understood. The criteria that define sites permissive for amber suppression using various tRNA/aaRS pairs have been identified in bacteria [35], but such information is currently lacking for eukaryotic cells. Thus, multiple positions within the POI need to be explored. In the case of viruses, the introduced modification should ideally provide high particle yields and have no effect on virus infectivity. Impairment of replication competence might influence the process being studied, and lower virus titers limit downstream applications. Additional restrictions may limit the options in the case of viral structural proteins, whose multifunctional nature renders them generally intolerant towards genetic modifications. This fragility may even manifest itself upon minimally-invasive modification by a single click-labeled ncAA. For example, a comprehensive mutational analysis (introducing natural amino acids) of the notoriously genetically fragile CA protein of HIV-1 revealed that ~ 70% of point mutations tested severely impaired or abolished viral infectivity [36]. On the other hand, a large database derived from mutational analyses, as well as structural information, is available for many viruses to aid the selection of promising target sites. 

#### 2.4.2. Detection Sensitivity

Viruses are small structures, whose detection against the cellular background may be challenging, in particular in live-cell experiments requiring signal quantitation. Therefore, in addition to labeling efficiency, the copy number of the POI within the viral structure of interest is a relevant parameter. Viral capsids are multimers of structural proteins; depending on the virus, they may comprise many monomers of a given protein (e.g., ~ 1500 CA molecules per HIV-1 capsid). AAV, a small virus whose capsid is formed from only 60 monomers, could be successfully detected in infected cells upon GCE/click labeling [28]. This exemplifies the possibility of imaging very small viruses with a low number of capsomers, at least upon in vitro click labeling of purified particles. Click-labeled HIV-1 Env on viral particles, where only 21–45 molecules are present per virion, was below the limit of microscopic detection, while the protein could be readily visualized at the surface of producer cells [32]. In principle, sensitivity may be increased by including more than one label per POI, but it needs to be kept in mind that each additional position targeted by GCE/click labeling makes the system less efficient and increases the invasiveness of the approach. 

#### 2.4.3. Incomplete Suppression

The incorporation of ncAAs by amber suppression is usually incomplete in eukaryotic cells, resulting in a mixture of modified POI and a variant truncated at the site of the introduced stop codon. While this may be a problem for any POI, it is a particularly relevant concern in the case of viral structural proteins. First, virus production generally relies on high protein yields, with a balanced stoichiometry between different virion components. Inefficient amber suppression may thus result in deficient particle formation by reducing the intracellular concentration of a structural protein below a critical value or by skewing the balance between different building blocks of the viral particle. Second, since these proteins need to assemble into regular structures, the probability that a truncated version may interfere with the assembly, and thus be dominant negative, is high, and may need to be experimentally assessed. 

The efficiency of amber suppression depends on numerous variables including (i) the protein of choice, (ii) the cell type used, (iii) the method to deliver the bioorthogonal tRNA/aaRS pair and resulting expression levels of those components, (iv) the cytosolic concentration of ncAA, and (v) the sequence context of the introduced amber codon within the mRNA. The relative contribution of each of these factors is not fully understood [10]. However, it is clear that high expression levels of orthogonal tRNAs [37], cytosolic localization of aaRS [38] and high intracellular concentrations of ncAAs [39] promote production of the engineered protein. An elegant way to increase suppression efficiency was described by Schmied et al. [40], who targeted the eukaryotic release factor eRF1 that terminates translation, and thus competes with the ncAA incorporation machinery at the stop codon. Expression of the dominant negative mutant eRF1(E55D) in the protein-producing cell shifts the balance in favor of amber suppression [40]. The application of this strategy boosted amber suppression efficiency ~fourfold in the case of the Env glycoprotein of HIV-1 [32]. 

#### 2.4.4. Choice of Cell Line

It should be noted that, to date, optimized GCE procedures have mainly been developed for standard cell lines that yield high transfection efficiencies and high expression levels of the amber suppression system and the POI (e.g., HEK293T). Such cell lines are perfectly suited for the production of engineered virus particles for subsequent infection experiments, i.e. for generation of virus samples for studying early viral replication steps. In contrast, the application of GCE/click labeling to analyses of later stages of the replication cycle requires labeling of viral proteins within the virus-producing cell, which is considerably more difficult. In this case, studies should ideally be performed on cell types that closely mimic the natural host cell of the respective virus, preferably primary cells, which are often refractory to transfection and much more challenging to manipulate. In this case, the potential effects on experimental results by alteration of cellular pathways and cytotoxic effects of the GCE also need to be considered. Implementing GCE/click labeling to study viral spread in more complex systems, e.g., organoids, would be an even more challenging endeavor. 

#### 2.4.5. Low Yield of Engineered Virus

Besides incomplete suppression, the fact that it is necessary to provide all components of the GCE system together with the virus in the same cell is an obstacle to the production of high virus yields. In the frequently used co-transfection approaches, the majority of the cell population needs to be efficiently co-transfected with one or more viral expression constructs, plasmids expressing aaRS and (multiple copies of) tRNA, as well as possibly a plasmid expressing eRF1(E55D) [40], in suitable stoichiometry to ensure efficient virus formation. Stable cell lines expressing at least part of the GCE components (e.g., [41]) would be an ideal solution, but sustaining high-level expression of the GCE system is challenging due to the interference of efficient stop codon suppression with cell viability. Efficient gene transfer methods using virus-derived vector systems present an alternative, also providing more flexibility regarding the cell type used. For this, several virus-derived gene transfer vectors are being explored, including vectors derived from adenoviruses [42], lentiviruses [43], AAV [44] and baculoviruses [45]. With their large coding capacity and ability to efficiently transduce different cell types (e.g., primary cells including mouse embryonic fibroblasts and rat cardiac fibroblasts, cultured neurons, as well as mouse embryonic stem cells), baculoviral vectors are a particularly attractive possibility for experiments in tissue culture with cell types which are difficult to manipulate [45].

#### 2.4.6. Unwanted Amber Suppression and Click Labeling

It needs to be taken into account that amber suppression is not specific to the codon that is experimentally introduced at the chosen site. All amber stop codons present in the cell in a suitable sequence context may, in principle, be recognized, resulting in the expression of elongated versions of the respective proteins. These versions may have no or altered functionality, or even exert dominant negative effects, resulting in the deregulation of cellular pathways and impairment of cell viability. Although the strategy is surprisingly well tolerated by cells in principle, it is bound to have pleiotropic and toxic effects, particularly when suppression efficiency is high. Moreover, amber suppression and click labeling of host cell proteins will lead to increased background labeling. Both cytotoxicity and background labeling may hamper the application of GCE and click labeling for the study of intracellularly labeled viral proteins by affecting the viral replication cycle and impairing sensitive detection of viral structures, respectively. Since the production of viruses in tissue culture is completed within a few hours to a few days post transfection, and the state of the producing cells should not affect the experimental outcome upon infection of untreated target cells, these considerations are less relevant when engineered viruses are used in analyses of the early replication steps.

In addition, viral open reading frames also terminate with stop codons, which may result in unwanted modification and labeling of viral proteins other than the POI. In both cases, virologists have some strategic advantage compared to cell biologists. The use of purified engineered viruses for the infection of native target cells circumvents potential toxic effects of the GCE procedure on the host cell. This advantage does, however, not apply to investigations of the late phase of viral replication cycles, where proteins need to be engineered, labeled and analyzed in the producer cell. Furthermore, viral genomes are often small and readily manipulated using standard molecular biology methods. Thus, non-targeted amber stop codons of viral genes might be converted to ochre or opal codons to avoid recognition by the GCE system. Since small genomes are frequently characterized by overlapping open reading frames or the coincidence of genes with regulatory nucleic acid sequences, the effect of such alterations on infectivity needs to be tested. Of particular interest for future studies is the orthogonal incorporation of two different ncAA carrying two different fluorophores or other functional groups, to allow, for example, for smFRET analyses. This advancement has been pioneered in *E. coli* [46] and is still under development for cell biological and virological applications.

#### 2.4.7. Choice of ncAA and Fluorophore

Since a small molecular footprint and suitable chemical properties of the modification may be crucial to retaining virus infectivity, the size of the ncAA and, more importantly, the size and chemical nature of the fluorophore and the length of linker between the two moieties have to be considered. Their effect on infectivity needs to be assessed and optimized; it may also be necessary to adjust the balance between labeling efficiency and infectivity by partial labeling. 

The chemistry of the ncAA and clickable dye are also of relevance for measurements within virus-producing cells, in particular for live-cell experiments. A high rate constant of the click reaction is of fundamental importance when labeling intracellular POI for live-cell observation. Furthermore, low rate constants necessitate high dye concentrations to achieve reasonable labeling efficiencies, which will also promote unspecific attachment of dye to intracellular membranes and proteins. The resulting background may impair the sensitive detection of individual viruses or subviral structures. In addition, side reactions of ncAAs bound to aminoacyl-tRNA synthetase may occur, requiring extensive and prolonged washing of samples to remove non-incorporated ncAAs [47]. These concerns are less relevant when GCE modified viruses are purified and labeled in vitro.

Whereas a wide selection of organic fluorophores is available for labeling viral surface proteins, choices for capsids of enveloped proteins or for viral proteins within a producer cell are more limited. The cell-permeable, fluorogenic, near-infrared dye silicon rhodamine (SiR; [48]; Figure 3d) and its derivatives, suitable for advanced microscopic applications, have set a paradigm. While SiR has been proven to be invaluable for live-cell labeling strategies, it has the disadvantage that it is a substrate for cellular efflux pumps [48], limiting its effective intracellular concentration, which, in turn, reduces the overall labeling efficiency (independent of the labeling strategy utilized). More fluorogenic, cell-permeable dyes are being developed, which may circumvent this limitation. The efflux of SiR does not present a concern for extracellular labeling of genetically-engineered viruses.

#### 2.4.8. Effect of Virus Replication on GCE

While GCE may affect processes in the viral life cycle under investigation, the opposite should also be considered. Viruses replicate by reprogramming the biosynthetic pathways of the host cell to favor the production of virus components, which, in turn, may interfere with the functionality of the introduced GCE system. Frequently used viral strategies include rapid and efficient host cell gene transcriptional or translational shutoff (exemplified by Rift valley fever viruses, as described below, or polioviruses, respectively), which could interfere with the required expression of components of the GCE system. It may thus be necessary to establish a careful balance between the expression of GCE components and the virus with respect to the level, as well as the timing, of expression. 

## 3. Click Labeling of Nucleic Acids Allows Detailed Analyses of Viral Replication

### 3.1. Viral Replication Sites

During a significant part of their replication cycle, viruses do not exist in the form of morphologically-distinct virus particles, but either as subviral nucleoprotein complexes, as genetic information only, or as newly-synthesized virion components. Detecting viral nucleic acids is therefore a central objective for scientists who want to image virus-cell interactions. Unlike eukaryotic cells with a default dsDNA genome, viruses store their genomic information in different forms: RNA or DNA, single or double stranded, segmented or as a single molecule. While dsDNA viruses have the ability to rely on the host cell machinery for genome replication, all other viruses have developed specific replication strategies, as well as strategies to convert genomic information into mRNA encoding viral proteins. Viruses can be classified according to their replication and transcription strategy (“Baltimore scheme” [19]). Most dsDNA viruses (except for the poxvirus family and other nucleocytoplasmic large DNA viruses) replicate their genome in the nucleus with the help of cellular DNA polymerases, whereas the replication sites of most RNA viruses are in the cytoplasmic area (with the notable exception of IAV and other orthomyxoviruses).

Viruses—especially those that replicate inside the cytosol—face some serious difficulties. Host cells have evolved sensor molecules that detect foreign nucleic acids and trigger intrinsic or innate antiviral defense mechanisms [49]. Furthermore, viral components need to be concentrated within the large volume of the cell to support high-level replication [50]. The evolutionary solution for these problems comes in the form of virus-induced replication compartments called viral factories, inclusion bodies or virosomes [51]. In many cases, these compartments are generated by remodeling intracellular membranes (e.g., from the endoplasmic reticulum, Golgi apparatus or mitochondria), forming invaginations or double-membrane vesicles that define and protect the viral replication site [50,52,53] Some viruses (e.g., the rabies virus and vesicular stomatitis virus) form membrane-less replication compartments, which may segregate from their surroundings by liquid-liquid phase separation [54,55]. Nuclear replication compartments of DNA viruses may be associated with changes to different nuclear domains, e.g., promyelocytic leukemia nuclear bodies (PML-NB) [51].

### 3.2. Site-Specific Nucleic Acid Labeling

A large number of studies detecting viral RNA or DNA via fluorescence in situ hybridization (FISH) have contributed to our understanding of basic virus biology. However, this technique can only be applied to fixed samples, and depends on the establishment of efficient and specific probes. The harsh treatment of samples required for FISH is often incompatible with immunofluorescence (IF) staining, and extensive extraction within the cytoplasm may render FISH non-quantitative, thereby underrepresenting subviral structures [24]. Most importantly, in many cases, FISH cannot distinguish between incoming genomes and de novo synthesized nucleic acids resulting from replication or transcription. Direct chemical labeling of incoming or newly-transcribed nucleic acids has the potential to overcome these limitations.

As described above, bioorthogonal building blocks can be site-specifically integrated into proteins by expanding the genetic code. The site-specific incorporation of bioorthogonal handles into nucleic acids is considerably more challenging, however. To date, the site-specific expansion of the genetic alphabet has only been achieved in *E. coli* by using two unnatural nucleotides. These form a third, unnatural base pair (UBP) besides the natural G-C and A-T, based on hydrophobic interactions [56]. Very recently, it was shown that such UBPs can be retained during in vivo replication [57] and can be transcribed into mRNA and tRNA. Furthermore, unnatural codons were decoded at the ribosome to incorporate ncAA into a model reporter protein [58]. However, this promising technology is still in its infancy, and is not yet ready for virological application.

### 3.3. Stochastic Nucleic Acid Labeling

In contrast, stochastic metabolic incorporation of modified nucleotides into viral genomes or replication intermediates has been successfully employed to elucidate virus biology. Although such approaches do not discriminate between viral and cellular nucleic acids, the viral target molecules may be distinguished based on their unique intracellular localization and/or co-localization with known viral markers. The first approach was based on bromodeoxyuridine (BrdU) incorporation, followed by IF detection of incorporated BrdU to define actively replicating viral genomes. As early as 1988, this method provided the first evidence that DNA viruses like the herpes simplex virus 1 (HSV-1) form replication compartments and recruit cellular replication machinery [59]. A major disadvantage of this methods is that, similar to FISH, it requires extensive denaturation (acid treatment or boiling of samples) to make the DNA accessible to the BrdU-specific antibody.

**Figure 5:**
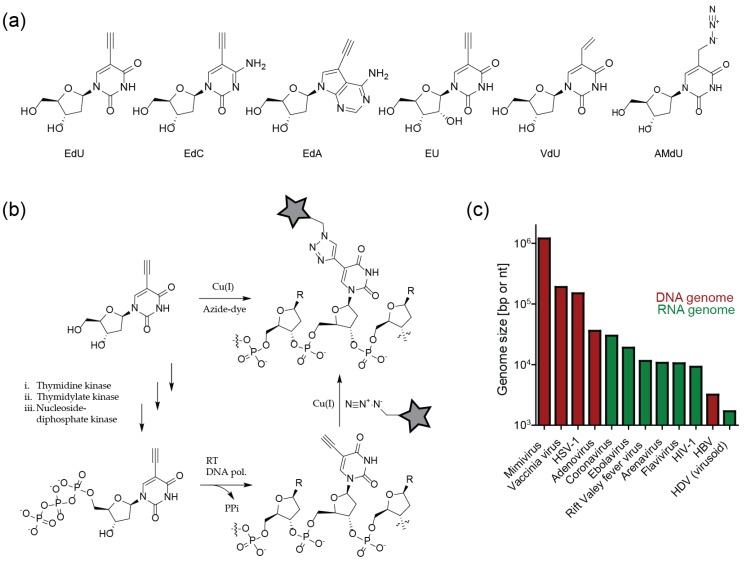
Labeling of viral nucleic acids by metabolic incorporation of unnatural nucleosides and click labeling. (**a**) Unnatural (deoxy)ribonucleosides suitable for click labeling approaches. (**b**) Intracellular phosphorylation and incorporation of an unnatural nucleoside/nucleotide and ligation to a fluorophore via CuAAC. (**c**) Sizes of exemplary viral genomes.

These limitations were overcome by bioorthogonal nucleosides bearing a small alkyne modification. This modification is readily accepted by nucleoside kinases, required for the intracellular transformation of nucleosides into the corresponding nucleotide, and by many nucleic acid polymerases. The prototype analog for this purpose, 5-ethynyl-2′-deoxyuridine (EdU), was described in 2008 by Salic and Mitchison [60], and is widely used in virology until today. This important contribution was followed by synthesis of other alkyne derivatives, e.g., deoxy-5-ethynylcytidine (EdC) [61] or 7-deaza-7-ethynyl-2′-deoxyadenosine (EdA) [62]. Furthermore, the ribonucleoside equivalent of EdU, 5-ethynyl-2′-uridine (EU) was developed to label nascent RNA [63] (Figure 5a). 

It should be noted that nucleotide analogs are substrates for cellular polymerases and other metabolic enzymes, and thus may exhibit cytotoxic effects upon prolonged incubation. Induction of cell cycle arrest, necrosis and general genome instability have indeed been described for the widely-used compound EdU, with IC_50_ values in the 1–10 µM range determined for different cell lines upon 72 h incubation [64]. This prompted the development of less toxic derivatives. F-*ara*-EdU, an EdU derivative with deoxyribose replaced by a D-arabinosyl (2′*S*) configuration with a fluorine substituent at the 2′-position, exhibited several orders of magnitude higher IC_50_ values in tissue culture compared to EdU [64]. The derivative EdC also has been described to be less toxic than EdU when incubated together with thymidine, which suggests that metabolic pyrimidine pathways may be partly responsible for cytotoxicity [61]. Based on this assumption, the purine nucleoside EdA was developed, which displayed ~ 10 fold higher IC_50_ values than EdU in tissue culture. In contrast to EdU, the IC_50_ value of EdA did not change dramatically upon prolonged incubation, indicating a non-genotoxic mode of action [62]. Many virological experiments are completed on a short time scale, where such cytotoxic effects may not be manifest. In general, the influence of nucleoside analog cytotoxicity on a planned experiment will depend on the compound, cell type, virus and time scale, and requires careful consideration.

### 3.4. Sensitivity of Viral Genome Detection

All of the compounds described above can be ligated to azide-coupled fluorophores using CuAAC (Figure 5b). Since Cu(I) treatment by itself is highly toxic, the method is restricted to fixed samples. On the other hand, the partial extraction of cells mediated by the harsh treatment favors efficient click labeling. The resulting enhanced signal-to-noise ratio makes it possible to detect relatively small viral genomes.

Signal intensities that can be achieved by metabolic labeling strategies depend on the size of the viral genome and the concentration of genome molecules at a given site. Virus families do not only differ greatly in morphology and replication strategy, but also in genome size; genome lengths range from little over 10^3^ nt (HDV) to over 10^6^ bp for members of the order *Megavirales* (giant viruses, e.g., mimivirus) (Figure 5c). Initial studies employing alkyne modified clickable nucleosides thus focused on HSV-1 [65], which carries a comparatively large dsDNA genome of 1.5 × 10^5^ bp and forms distinct replication compartments within the nucleus. Replication compartments of AdV (3.6 × 10^4^ bp) were also detected by click-based approaches [65]. The lack of proofreading in RNA synthesis limits the size of RNA virus genomes, making them more difficult to detect. The large ssRNA genomes of coronaviruses (~ 3 × 10^4^ nt), however, have been successfully visualized by metabolic labeling and click chemistry [66]. The detection of individual nascent reverse transcribed HIV-1 cDNA (2 molecules of ~ 10^4^ bp) in the cytosolic area of infected cells upon EdU incorporation and/click labeling has also been accomplished [67,68,69]. However, detection of these weak signals by stochastic labeling required the suppression of the host cell DNA synthesis using aphidicolin; virus-derived signals at or within the nucleus could only be visualized in non-dividing cells [68].

In the following sections, we will summarize some important contributions to the understanding of DNA virus, retrovirus and RNA virus biology gained by imaging of click-labeled nucleic acids.

## 4. Insights into Virus Biology Obtained Using Click Labeling of Viral Nucleic Acids

### 4.1. DNA Viruses

In a seminal study, Wang et al. [65] showed that alkyne-modified nucleosides can be employed to visualize DNA virus replication compartments within the nucleus of infected cells, as well as individual incoming viral particles labeled in the producer cell. Comparative analyses of HSV-1, AdV and the poxvirus vaccinia revealed differences in nucleoside analog preferences. While EdU, the slightly less toxic derivative F-ara-EdU, EdA and EdC could all be incorporated into the AdV genome, signals for vaccinia virus were only detectable using EdU (and less efficiently with EdC). In contrast, EdA and EdC yielded the strongest signals in the case of HSV-1, with F-ara-EdU being slightly weaker [65]. These pronounced differences might not only be explained by the different selectivities of the respective viral DNA polymerases, but may also result from differential nucleoside phosphorylation efficiencies within the specific host cell types. Furthermore, many viruses encode nucleoside or nucleotide modifying enzymes that either alter (deoxy)nucleoside triphosphate levels (e.g., HSV-1 thymidine kinase (TK), which catalyzes monophosphorylation, or dUTPase, which hydrolizes dUTP), or modify DNA after replication (e.g., uracil DNA glycosylases (UNG) that cleaves the *N*-glycosidic bond of misincorporated dUMP from DNA).

#### 4.1.1. Recent Insights into Herpes Simplex Virus Biology are Fueled by Click Chemistry

Within the last few years, scientists studying HSV-1 have accumulated a growing body of data generated by employing click labeling of viral DNA. The data obtained using single virus imaging corroborated results from ensemble measurements, and provided novel insights into HSV-1 replication as well as dsDNA viral biology in general. After HSV-1 fuses with the host cell membrane, the capsid is transported to the nuclear pore complex (NPC), where uncoating of the viral capsid and nuclear transport of the dsDNA genome occurs. Within the nucleus, HSV-1 creates replication compartments (RC) that provide an ideal environment to synthesize large amounts of progeny DNA, transcribe mRNA to produce viral proteins, and assemble newly-formed capsids with viral dsDNA. RC are often found adjacent to nuclear organizing structures, one of them being PML nuclear bodies (PML-NB) that are implicated in the cellular defense against viruses.

Since the incorporation of EdU into HSV-1 DNA was found to be inefficient [65], Dembowski et al. [70] reasoned that the activity of the viral dUTPase and UNG enzymes may limit EdU incorporation. Knock-out of both genes in the HSV-1 genome indeed resulted in higher labeling efficiency of viral particles. The presence of the bioorthogonal handle on the viral DNA enabled the authors to attach different functionalities in order to complement biochemical analysis and imaging [70]. Cells were infected with EdU-carrying particles, and viral DNA was pulled down upon click coupling to biotin-azide. Mass spectrometric analysis of viral DNA-associated proteins revealed over 200 cellular and viral proteins (e.g., transcription factors, cellular DNA replication and repair machinery, RNA processing proteins and chromatin remodeling factors) selectively recruited to HSV-1 RCs. Intriguingly, abundant chromatin remodeling complexes, but no histones, were detected in these experiments. The underrepresentation of histones at viral replication sites was confirmed by imaging of click-labeled particles, indicating that HSV-1 DNA is not extensively chromatinized during replication [70]. In a follow-up study, the authors expanded on these findings by utilizing a similar strategy, but limiting an EdC pulse to 5–20 min; this enabled the selective incorporation of EdC into active HSV-1 replication forks. Different pulse times, together with pulse-chase experiments, revealed the dynamic binding of DNA repair proteins and transcription factors to the viral replication fork, and indicated the coupling of those processes to DNA replication [71]. Again, the results were confirmed by imaging upon clicking a fluorophore to incorporated EdC and observing colocalization of selected factors with HSV-1 RC [71]. Interestingly, nascent DNA moved outwards, indicating the existence of functionally-separated subdomains within the viral RC.

**Figure 6:**
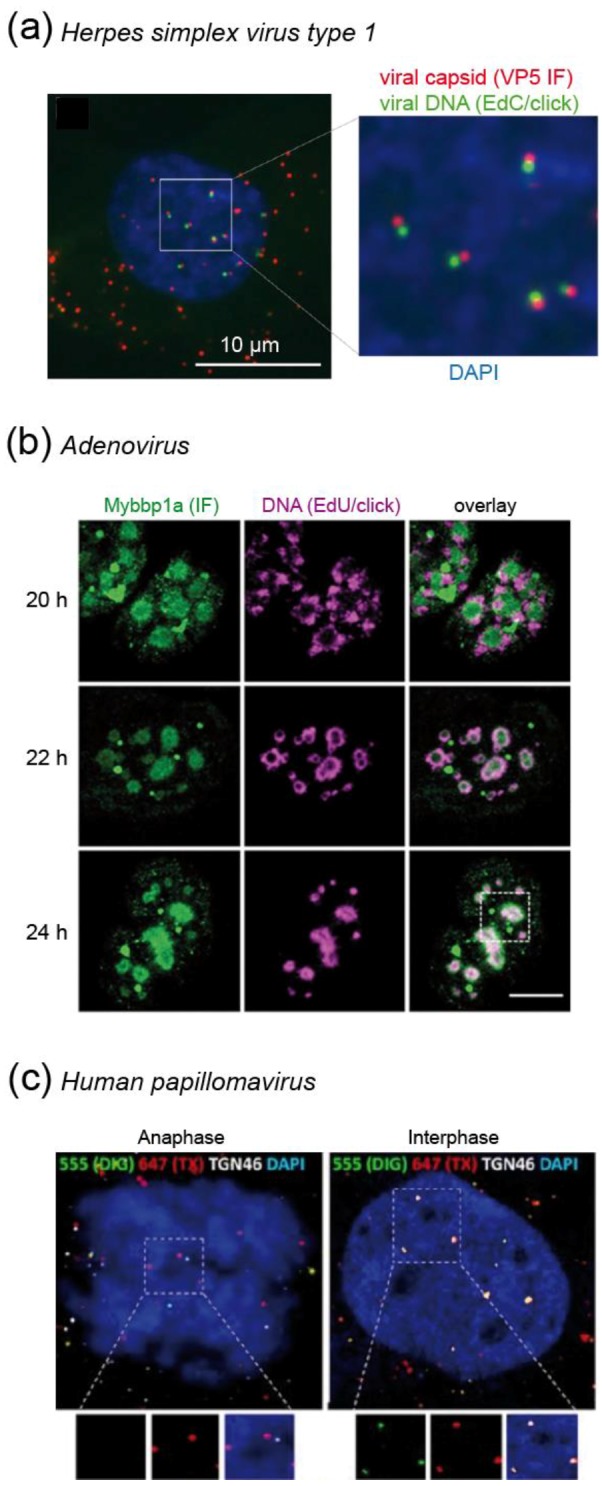
Examples for visualization of DNA virus replication stages. (**a**) Quantitative detection of HSV-1 uncoating. The maximum projection shows accessible viral genomes in the nucleus (green) still closely associated to viral capsids (red). Viral DNA contained in capsids in the cytosolic area is not accessible to the fluorophore (red signals only) [72]. (**b**) Pulse-chase labeling of nascent AdV DNA in the nucleus of virus producing cells shows relocalization of labeled DNA from the periphery to the core of viral replication centers from 20–24 h post infection [73]. (**c**) Differential accessibility of HPV DNA to fluorophores after mild (green) and harsh (red) detergent treatment of infected cells reveals that the incoming viral genome is protected in vesicles during anaphase (left panel; red signals only) and becomes accessible upon completion of mitosis (right panel; co-localization of green and red signals) [74]. Images were modified from the indicated references, with permission.

Sekine et al. [72] expanded on these findings with a detailed microscopic analysis of incoming HSV-1 replication dynamics. Cells were infected with particles carrying EdC incorporated into the viral genome, followed by click labeling of the entering particles with a functionalized Alexa dye. Strikingly, the viral DNA was only occasionally detected inside the cytoplasm of infected cells, while nuclear signals where readily detectable (Figure 6a). The differential labeling suggested that the DNA contained in the viral capsid was inaccessible to the fluorophore, while DNA ejected in the nucleus after viral uncoating at the NPC could be readily chemically modified. This assumption was confirmed by comparing staining of native virions with particles that had undergone in vitro induced uncoating [72]. Thus, in the case of HSV-1, the bioorthogonal DNA labeling strategy not only serves to detect genomes, but can also report on the state of the viral capsid. 

Foreign DNA within the cytoplasm rapidly triggers innate immune responses leading to interferon (IFN) production [49]. Protection of the incoming HSV-1 genome by the viral capsid mediates evasion from antiviral defenses until the particle reaches the nucleus. The uncoating step occurring at this site is accompanied by DNA decondensation, rendering the genome accessible for click labeling [72]. Since the DNA now also becomes accessible to innate immune sensors, e.g., the IFNγ Inducible Protein 16 (IFI16) or PML-NB, HSV-1 antagonizes these antiviral proteins by proteasomal degradation. Using EdU labeled HSV-1 particles that carried a knock-out of the respective antagonizing protein (ICP0-null mutant), Alandijany et al. [75] intriguingly observed rapid entrapment of HSV-1 genomes by PML-NB upon nuclear import. The authors elegantly demonstrated that the presence of incoming HSV-1 DNA within PML-NB by itself was not sufficient to induce a cellular IFN response, and IFI16 was not observed to colocalize with HSV-1 DNA at early time points [75]. A complementary study that amplified the IFI16 IF signal by a proximity ligation assay (PLA) with two different antibodies revealed that only very low levels of IFI16 localized to incoming genomes [76]. Most importantly, the saturation of IFI16, which is necessary to elicit a robust IFN response, only occurred after the replication of viral DNA [75]. These findings elucidated an elaborate dual strategy of the host cell to counteract HSV-1: the incoming viral genomes are entrapped to prevent replication; failure to prevent viral DNA replication results in shift to an antiviral state in order to curtail further viral spread. Of note, these studies were only made possible by the high sensitivity of the EdU click labeling strategy, which enabled researchers to analyze individual incoming particles under low multiplicity of infection (MOI) experimental conditions. The low expression levels of intrinsic host nucleic acid sensors can be rapidly saturated by infection at high MOI [75], which previously obscured the kinetics and temporal intricacies of the recruitment process.

Since dsDNA viruses generally replicate inside the nucleus and employ the host cellular machinery, they are also highly dependent on the cell cycle. Strikingly, HSV-1 infection with a very low MOI coincident with EdC pulse incorporation and click labeling of cellular DNA revealed that while HSV-1 infection blocks cellular DNA replication at the G1/S phase transition in the infected cells, cellular DNA synthesis was drastically enhanced in uninfected bystander cells [77]. This was attributed to a (yet to be identified) paracrine effector, which was stimulated by HSV-1 infection, and may prime neighboring cells for subsequent infection [77]. Another creative use of metabolic labeling and click chemistry took advantage of the fact that HSV-1 encodes its own thymidine kinase (HSV-1 TK), which is characterized by a more promiscuous substrate range compared to cellular TK. Due to selective phosphorylation by the viral enzyme, 2′-deoxy-2′,2′-difluoro-5-ethynyluridine (dF-EdU) is only incorporated into DNA in HSV-1 infected cells, and thus serves as a bioorthogonal marker for viral infection [78].

#### 4.1.2. Adenovirus Replication: Click Labeling Revealed a New Virus Induced Nuclear Substructure

Similar to the HSV-1 example, the click labeling of EdA/EdC labeled viral DNA could be exploited in the case of AdV to obtain information on the dynamics of the viral uncoating process [65]. In humans, AdV may cause various types of infections (eye, respiratory or gastrointestinal tract). They enter the cell via endocytosis. After escape from the endosome by activation of the viral membranolytic protein VI, particles traffic to the NPC, where the genome is released into the nucleus. Cell free particles carrying alkyne-modified genomes were refractory to click labeling. In contrast, signals were readily detected for the majority (~ 75%) of entering capsid positive complexes in the cytosol at 30 min post infection (when particles are assumed to be released from the endosome), indicating partial uncoating [65]. At 3 h post infection, only one third of capsid-containing viral particles remained associated with the viral DNA, indicating genome release. Of note, this detectable uncoating was completely blocked by leptomycin B, an inhibitor of nuclear export that also blocks docking of AdV particles to the NPC [65]. 

Once the AdV genome reaches the nucleus, it induces intranuclear RC. Komatsu et al. [73] showed that these can be separated into early spherical and late ring-like structures. By using EdU/click pulse-chase experiments, they observed that de novo synthesis of viral DNA occurs in the outer region of the late RC, followed by movement into the core of the compartment (which they termed Virus-induced Post-Replication (ViPR) body), where progeny viral genomes accumulate (Figure 6b) [73]. Histones were present on newly replicated genomes, but absent in ViPR bodies and in virions. Therefore, the authors speculated that restructuring of viral genomes occurs within the ViPR bodies before packaging and release to infect new cells [73].

#### 4.1.3. Human Papillomavirus Protects Its Genome by Special Transport Vesicles that are Delivered into the Nucleus During Mitosis

Human papillomaviruses (HPV) contribute to the etiology of many benign and malignant tumors, most notably to cervical cancer. The cellular entry of HPV is a complex process, involving endocytosis, endosomal acidification and activation of proteases, as well as transport to the trans Golgi network (TGN) [79]. Click labeling of viral DNA contributed to the characterization of the requirements and dynamics of the HPV entry process. For example, EdU-labeled particles were employed to demonstrate that HPV entry depends on the activity of the trypsin-like serine protease kallikrein-8 (KLK8); knockdown of KLK8 impaired intracellular trafficking of incoming virions to the TGN [80]. 

HPV forms RC that colocalize with PML-NB in established infections, where replication may be initiated. Unlike most DNA viruses that gain access to the nucleus through the NPC (e.g., HSV-1 or AdV), HPV requires nuclear breakdown during mitosis for nuclear transfer. This was first thoroughly analyzed by Aydin et al. [81] using an automated high-content RNAi screen. Besides other factors, the authors identified proteins that are important in the regulation of the cell cycle and mitosis. EdU click-labeling of viral genomes confirmed the dependence of HPV infection on nuclear envelope breakdown [81]. Of note, the authors initially used BrdU for the detection of viral genomes, but switched to EdU, because the detection via CuAAC was more sensitive. In a follow-up study, Broniarczyk et al. [82] analyzed the time required for HPV nuclear penetration. In cells synchronized in G2/M, EdU positive viral genomes associated with PML-NB inside the nucleus within only 1 h post infection, much faster than previously thought. In contrast, EdU-labeled HPV DNA accumulated in the cytosol in cells arrested in the G1 phase [82].

Since HPV particles lose a major part of the capsid during endocytic trafficking [79], it remained unclear how HPV protected its genome from cytosolic antiviral defenses during trafficking from the TGN to the nucleus. DiGiuseppe et al. [74] used a sequential double click strategy of EdU tagged incoming viral genomes to determine that the HPV genome is found within a protective vesicle throughout the whole process of mitosis. They first partially permeabilized the plasma membrane and early endosomes using low digitonin concentrations, followed by click labeling with the membrane impermeable Alexa Fluor 550 (AF550)-azide dye. Subsequently, samples were fully permeabilized followed by click labeling with AF647-azide to visualize previously protected genomes. Only after mitosis and reformation of the nuclear envelope, EdU-labeled viral genomes became accessible to partial permeabilization, resulting in AF550 and AF647 double-labeled signals (Figure 6c) [74]. Validating their findings by careful controls and biochemical experiments, the authors thus uncovered a novel vesicular transport mechanism of cargo into the nucleus during mitosis. Vesicles can apparently exist for some time within the nucleus. While the origin of the vesicles remains to be established, the authors speculated that HPV hijacks vesicles during the fragmentation of ER and TGN in mitosis [74].

#### 4.1.4. Visualization of Vaccinia Virus DNA in Viral Replication Factories and Incoming Virions

The vaccinia virus and other poxviruses belong to the group of nucleocytoplasmic large DNA viruses, which are characterized by large viral replication factories in the cytoplasmic area of the host cell. Wang et al. demonstrated that de novo synthesized vaccinia virus DNA can be efficiently labeled by EdU incorporation and click labeling within these cytoplasmic replication compartments. This finding was exploited by Yakimovich et al. [83] to characterize the effects of bisbenzimide dyes on the viral life cycle. The authors had identified molecules from this class as specific inhibitors of vaccinia virus infectivity, and thus, potential lead compounds for anti-poxvirus drug development. They observed that the compounds affected intermediate and late viral gene expression, which requires viral DNA replication. Bright cytoplasmic EdU signals originating from vaccinia virus replication compartments were significantly reduced in the presence of the tested bisbenzimides, showing that these compounds indeed act by blocking viral DNA replication [83]. 

EdU/click labeling of vaccinia genomes was further employed to develop a direct microscopic readout for poxvirus uncoating by infecting cells with double labeled vaccinia virions carrying EdU modified DNA and an eGFP labeled viral core protein, and using separation of the EdU/click signal from eGFP labeled capsids as a measure for genome release [84]. Using this readout, Kilcher et al. [84] demonstrated that the viral ATPase D5, which they had previously identified as a candidate protein in a siRNA screen, is an important vaccinia virus uncoating factor. Two spots of D5 were observed to be associated with the long sides of incoming viral cores by SIM, suggesting that these D5 foci might serve as portals to release the viral DNA [84].

### 4.2. Retroviruses

**Figure 7:**
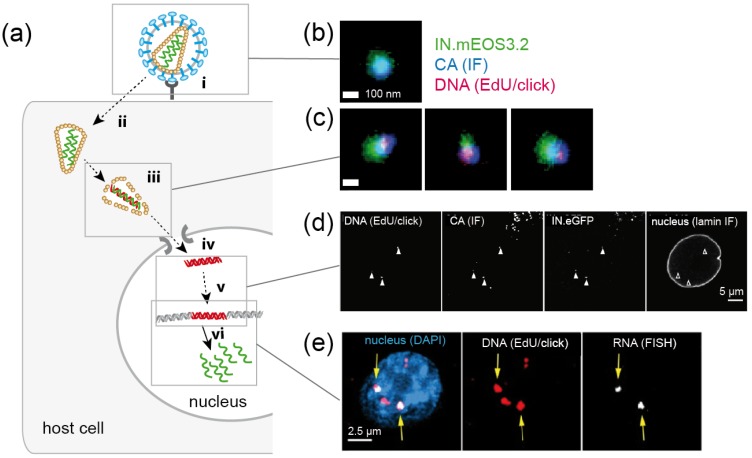
Imaging of HIV-1 replication complexes in primary human macrophages (**a**) Simplified schematic representation of post-entry replication stages of HIV-1. The virus attaches to receptor and co-receptor molecules at the host cell surface (i), and the viral Env protein mediates fusion of the viral envelope with a host cell membrane, resulting in cytosolic entry of the conical capsid (ii). Reverse transcription of the viral genome, transport to the NPC and capsid uncoating (iii) are spatially and temporally intertwined; this part of the replication cycle is poorly understood. A viral nucleoprotein complex is transported through the NPC into the nucleus (iv), where the viral cDNA is integrated into the host cell genome (v) and serves as a template for transcription of viral RNA (vi). Green, RNA; red, DNA. (**b**,**c**) Nanoscopic visualization of HIV-1 particles (**b**) and cytosolic subviral complexes (**c**). Three-color PALM/dSTORM images reveal co-localization of nascent HIV-1 DNA in reverse transcription complexes with the viral proteins CA and integrase [68]. (**d**) HIV-1 CA is detected at viral reverse transcription complexes in the nucleus of infected primary macrophages by confocal microscopy [67]. (**e**) Newly transcribed HIV-1 RNA co-localizes with a subset of EdU/click labeled HIV-1 DNA in the nucleus (arrows) [69]. Images in (**b**–**e**) were modified from the indicated references, with permission.

After entering the host cell, retroviruses like HIV-1 convert their ssRNA genomes into dsDNA. This so-called reverse transcription is a prerequisite for the integration of the proviral DNA into the host genome and for productive infection. Retroviral reverse transcription is initiated within the viral capsid, and apparently proceeds during intracellular transport and the uncoating of subviral complexes. All processes in this early post-entry phase appear to be temporally and spatially linked, and have to be tightly controlled to allow for productive infection. Since the post-entry phase is not only the least well understood part of the retroviral replication cycle, but also the target for a number of intrinsic antiviral host factors, it has become a focus of intense investigation in the past decade (Figure 7) [85,86].

#### 4.2.1. Using Click-Labeled Viral RNA to Monitor HIV-1 Uncoating

In principle, SVT using virions click-labeled with different fluorophores at different components would be an ideal approach to study spatial and temporal relationships between reverse transcription, trafficking and uncoating. While labeling of the viral capsid remains challenging (see above), click labeling of HIV-1 genomic RNA, as well as of newly reverse-transcribed DNA, has been achieved in fixed samples (Figure 7b–e) [67,68,69,87,88]. As described above for DNA viruses, differential accessibility of an EU-modified HIV-1 genome within entering subviral structures has been characterized to obtain information on loss of the closed conical capsid structure. Infecting HeLa-derived cells with EU-modified virions, Xu et al. [88] observed that the viral RNA was accessible to an azide-coupled dye only subsequent to virus-cell fusion followed by an initial core opening step, arguing for biphasic uncoating. RNase A could not access RNA within immobilized particles in vitro, indicating protection of the packaged viral genome from cytosolic nucleic acid sensors and degrading nucleases [88].

#### 4.2.2. Detection of HIV-1 Reverse Transcription Complexes by EdU/Click Labeling

A major difficulty in the use of SVT for studying post-entry processes is that not all entry events are productive. Depending on the virus and the host cell type, a significant proportion, or even the vast majority, of particles entering a cell may end up in non-productive pathways, e.g., endosomal degradation. In the case of HIV-1, the detection of nascent reverse transcription products by EdU incorporation and click labeling has been employed to identify particles undergoing productive reverse transcription [68]. Since retroviruses perform DNA synthesis outside of the nucleus, and at least lentiviruses such as HIV-1 are able to infect non-dividing cells that do not undergo de novo cellular DNA synthesis, the sensitivity of the metabolic labeling procedure was sufficient to detect individual HIV-1 reverse transcription products (<10 kb) within subviral complexes. Although the detection of viral DNA by itself does not prove the infectivity of a given particle, it demonstrates that the respective particle is capable of reverse transcription. Furthermore, while it is not quantitative, the degree of labeling provides an estimate for the progression of the reverse transcription process. 

Using this approach combined with IF detection of the HIV-1 capsid protein (CA), Peng et al. [68] observed extensive association of CA signals with reverse transcription competent particles in the cytosol (Figure 7c), confirming the initiation of reverse transcription within a capsid or capsid-derived structure. Unexpectedly, strong CA signals also colocalized with subviral complexes in the nucleus in certain cells types, most notably primary human macrophages (Figure 7d) [67,68], suggesting that the viral capsid (or a derived structure with a similar number of CA molecules) had passed the NPC. This observation is intriguing, since the distal ring diameter of the NPC was found to be ~ 40 nm [89], while the broad end of the conical HIV-1 capsid is around 60 nm wide [90]. Further analyses are required to confirm this finding, to compare different physiologically relevant host cells and to characterize the potential mechanism of nuclear import. 

A recent, more detailed investigation of HIV-1 replication dynamics in primary macrophages by Bejarano et al. [67] combined the EdU imaging-based approach with virological measurements. The authors identified delayed reverse transcription, caused by the depletion of the available dNTP pool by the host restriction factor SAM and HD domain-containing protein 1 (SAMHD1), as the main bottleneck responsible for the slow replication of the virus in this host cell type [67]. Quantification of EdU/click signals associated with particles at different intracellular localizations revealed higher signals on nuclear complexes compared to cytosolic particles, and in particular, to those detected near the nuclear envelope. This observation led to the hypothesis that reverse transcription may enhance the efficiency of nuclear entry and/or that—contrary to the current belief—HIV-1 reverse transcription may only be completed after nuclear import in this cell type [67]. 

These studies are complemented by work from Stultz et al. [69], who demonstrated viral RNA transcription from EdU positive HIV-1 cDNA in the nucleus (Figure 7e). Since non-integrated retroviral DNA is rapidly chromatinized and silenced inside the nucleus [91], the detected replication sites most likely represent integrated proviruses. The demonstration that EdU tagged HIV-1 DNA can be integrated, and serves as a template for RNA transcription, provides clear evidence for the minimally-invasive nature of the bioorthogonal modification.

Of note, some retroviruses could potentially increase the efficiency of modified deoxynucleotide incorporation into their genomes by degrading SAMHD1. The triphosphohydrolase SAMHD1 converts dNTPs to deoxynucleosides and inorganic phosphate, depleting intracellular dNTP pools, and thus impairing retroviral reverse transcription [92]. Some retroviruses, e.g., HIV-2 or simian immunodeficiency viruses encode the accessory protein Vpx which mediates proteasomal degradation of SAMHD1, and thereby increases dNTP levels. Depending on the specificity of SAMHD1 for the deoxynucleoside derivative used, this may also enhance intracellular levels of clickable dNTPs.

### 4.3. RNA Viruses

The vast majority of RNA viruses replicate within the cytosol, where specialized viral factories promote their replication. BrU labeling has been extensively used to visualize these compartments, but in recent years, the emergence of click chemistry has started to yield novel insights into the biology of some RNA viruses. The technology has, however, not yet been used as frequently as for DNA viruses. Most commonly, EU is employed to label de novo transcribed cellular RNA, resulting in extensive signals from nucleoli that harbor abundant ribosomal RNA as well as weaker cytosolic signals from exported mRNA [63]. The detection of small RNA virus genomes against this cellular background by metabolic labeling usually requires blocking of cellular RNA transcription using actinomycin D. 

**Figure 8:**
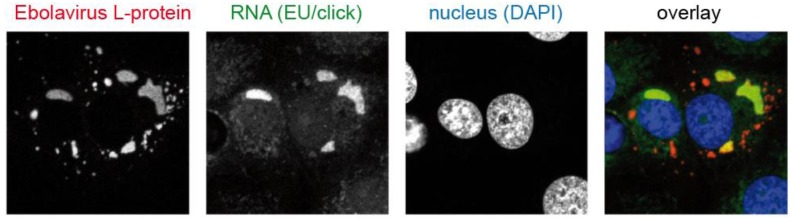
Visualization of ebolavirus replication compartments. Nascent viral RNA co-localizes with viral proteins in large cytosolic inclusion bodies. Modified from [93], with permission.

In 1967, several people became infected by a newly discovered virus named the “Marburg” virus (a (–)strand RNA filovirus) upon contact with infected monkeys. Initial light and electron microscopy analyses of infected cells revealed virus-induced cytosolic inclusion bodies of variable size and morphology. At the time, it was unclear if these structures represented a productive stage within the viral replication cycle, accumulations of surplus virus, inert aggregates of nucleocapsids, or other abortive structures. Much later, Hoenen et al. [93] demonstrated convincingly for the closely-related ebolavirus that these inclusion bodies are, in fact, the site of viral RNA replication by employing EU click labeling and imaging (Figure 8). Nascent RNA click signals co-localizing with the viral polymerase and nucleocapsid were exclusively detected in larger inclusion bodies, indicating that filovirus inclusion bodies undergo a maturation process, accumulating components for efficient viral RNA replication [93]. A combination of RNA click labeling with a correlative light and electron microscopy (CLEM) approach to compare the ultrastructure of RNA positive vs. negative replication compartments would be of great interest. 

The gradual development of viral factories has also been observed for coronaviruses (CoV). This virus family, which comprises pathogens causing respiratory tract infections in humans, is characterized by the largest known viral RNA genomes (up to ~ 4 × 10^4^ bp). Virus replication occurs within the cytosol forming membrane invaginations derived from the ER. Replication of their (+)strand RNA genome involves partially double-stranded RNA intermediates. The detection of dsRNA by IF was thus used as a surrogate for active viral replication. Hagemeijier et al. [66] used the mouse hepatitis virus (MHV) as a model to study coronaviral replication factories by EU incorporation and click chemistry. Surprisingly, while IF detection of dsRNA early post infection correlated with EU click signals, indicating nascent RNA synthesis, this correlation was lost at later time points, suggesting that factories lose their replicative capacity over time, and that the labeling of de novo synthesized RNA is necessary to identify sites of ongoing CoV replication [66]. For the CoV porcine transmissible gastroenteritis virus (TGEV), it was shown that EU click signals colocalize with the viral RNA-dependent RNA polymerase (RdRP) and the viral nucleocapsid protein [94]. This system was used to characterize the mode of action of a small molecule CoV inhibitor, which targets the viral RNP complex and blocks RNA replication as well as RdRP and nucleocapsid synthesis [94]. Other examples where EU/click labeling was successfully employed include chikungunya virus [95] and arenavirus replication-transcription complexes [96]. For members of the medically-important genus of *Flavivirus* (e.g., Dengue virus, Zika virus, yellow fever virus) and the related genus *Hepacivirus* (e.g., hepatitis C virus), systems for following virus replication by metabolic/click labeling are yet to be established.

A general viral strategy is to hijack metabolic pathways in order to redirect cellular resources towards the generation of new viral particles. One important mechanism involves the shutdown of host RNA transcription without affecting viral RNA replication (by using viral RNA-dependent RNA polymerases (RdRP) and specialized transcription initiation mechanisms). Metabolic incorporation of EU can be used to visualize host cell transcriptional shutdown during RNA virus infection. For example, this approach was employed to investigate the role of the non-structural protein s (NSs) of members belonging to the order of *Bunyavirales* as a pathogenicity factor [97]. Cells infected with Rift valley fever virus (RVFV), which is highly pathogenic in animals and can also cause severe illness in humans, showed decreased EU/click signals of cellular genomic DNA, indicating impaired transcription; this effect was also observed upon expression of NSs alone [98]. In contrast, the NSs protein of the related but less virulent Toscana Virus (TSV) had no effect on host cell transcription and EU-click levels [99].

### 4.4. Future Challenges and Opportunities

A disadvantage of the metabolic incorporation of modified nucleotides is that the strategy is not specific to the viral structure or process of interest, but detects all nascent DNA (or RNA) molecules. This can present significant difficulties for detecting signals from individual viral particles. The problem may be addressed via reduction of cellular background using small molecule inhibitors (e.g., aphidicolin for host cell DNA synthesis; actinomycin D for host cell transcription). However, impairment of host cell functions by drug treatment may affect experimental results. Furthermore, aphidicolin also blocks DNA polymerases of some viruses (e.g., HSV-1 or vaccinia virus). Nucleoside analogs that are preferential substrates for the viral polymerase of interest, and are thus specifically incorporated, would overcome this obstacle. 

Expanding the concept further, the site-specific incorporation of unnatural base pairs would be highly beneficial. To date, this has not been achieved in eukaryotes, but the first results from prokaryotic cells [56,57,58] are promising. This approach would not only increase the number of unnatural amino acid residues that could be encoded, but it would also allow incorporation of fluorophores at defined nucleotide positions and the application of super-resolution microscopy to study the ultrastructure of DNA or RNA within viral particles. Structural rearrangements during e.g., reverse transcription of retroviruses could be visualized and correlated to distinct steps during this complex process. Expanding the EdU-mediated detection of retroviral reverse transcription and pre-integration complexes from HIV-1 [67,68,69] to other retroviruses, comparing the post-entry behavior of lentiviruses that have the ability to infect non-dividing cells to that of simple retroviruses requiring nuclear breakdown during mitosis would shed further light on the complex requirements for retroviral replication.

In order to advance from fixed samples to live cell observations of viral nucleic acids, strategies involving the introduction of nucleotide sequences recognized by reporter proteins fused to FPs have been established (e.g., [100,101,102]). However, these approaches mostly require genetic modification of the viral genome with large and often repetitive sequences. A simple and rapid method that allows the use of wild type virus together with recently developed live-compatible click reactions (e.g., inverse electron-demand Diels-Alder reactions) is still missing. Such systems would not only significantly advance our understanding of DNA and RNA dynamics in virus replication; they would also enable ultrastructural investigation of RNA and DNA containing subviral complexes by CLEM and cryo-CLEM, which is currently hampered by cellular extraction and destruction of ultrastructure through CuAAC. Nucleosides bearing vinyl- [103] or azidomethyl groups [104] have been developed with the aim of allowing copper-independent click labeling of DNA under live cell conditions. Currently available derivatives are characterized by slow reaction kinetics, which impede the observation of dynamic processes. Furthermore, the efficient click labeling of these analogs requires the denaturation of samples with hydrochloric acid, precluding live cell applications. Novel nucleosides that can be efficiently click-labeled under live cell conditions with rapid kinetics would be beneficial not only for virological studies, but also for cell biology in general.

## 5. Conclusions

The past two decades have seen exciting developments in advanced fluorescence microscopy techniques and single molecule approaches, opening new possibilities for detailed investigations of virus-cell interactions at the single particle or single molecule level. By combining specificity, spatial precision and flexibility with respect to the choice of the click ligand, the minimally-invasive modification of virion components via click chemistry allows virologists to fully profit from these technical advancements and to complement imaging studies by biochemical analyses. While the click labeling of viral nucleic acids has already yielded numerous insights into virus biology, the exploitation of click chemistry for labeling viral proteins has just begun, and the extension of the approach to the investigation of viral lipids still lies ahead. In the coming years, improvements with respect to orthogonal multi-color labeling at multiple sites, stable GCE competent cell lines or even organisms and live-cell compatible systems can be expected, promising bright prospects for virus imaging.
